# Elucidating the Rate‐Limiting Processes in High‐Temperature Sodium‐Metal Chloride Batteries

**DOI:** 10.1002/advs.202201019

**Published:** 2022-04-11

**Authors:** Daniel Landmann, Enea Svaluto‐Ferro, Meike V. F. Heinz, Patrik Schmutz, Corsin Battaglia

**Affiliations:** ^1^ Empa Swiss Federal Laboratories for Materials Science and Technology Dübendorf 8600 Switzerland; ^2^ Laboratory of Renewable Energy Science and Engineering Ecole Polytechnique Fédérale de Lausanne Lausanne 1015 Switzerland

**Keywords:** molten salt batteries, sodium‐nickel chloride batteries, ZEBRA batteries

## Abstract

Sodium‐metal chloride batteries are considered a sustainable and safe alternative to lithium‐ion batteries for large‐scale stationary electricity storage, but exhibit disadvantages in rate capability. Several studies identify metal‐ion migration through the metal chloride conversion layer on the positive electrode as the rate‐limiting step, limiting charge and discharge rates in sodium‐metal chloride batteries. Here the authors present electrochemical nickel and iron chlorination with planar model electrodes in molten sodium tetrachloroaluminate electrolyte at 300 °C. It is discovered that, instead of metal‐ion migration through the metal chloride conversion layer, it is metal‐ion diffusion in sodium tetrachloroaluminate which limits chlorination of both the nickel and iron electrodes. Upon charge, chlorination of the nickel electrode proceeds via uniform oxidation of nickel and the formation of NiCl_2_ platelets on the surface of the electrode. In contrast, the oxidation of the iron electrodes proceeds via localized corrosion attacks, resulting in nonuniform iron oxidation and pulverization of the iron electrode. The transition from planar model electrodes to porous high‐capacity electrodes, where sodium‐ion migration along the tortuous path in the porous electrode can become rate limiting, is further discussed. These mechanistic insights are important for the design of competitive next‐generation sodium‐metal chloride batteries with improved rate performance.

## Graphical abstract

Electrochemical de‐/chlorination of nickel and iron model electrodes is studied in molten sodium tetrachloroaluminate at 300 °C in a planar cell geometry. While the transport of metal‐ions through the melt emerges as rate‐limiting process in both cases, nickel and iron electrodes exhibit different chlorination behaviors. This results in dissimilar rate capabilities and cycle life.

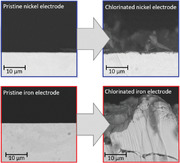

## 1 Introduction

Sodium‐metal chloride batteries, typically operated at 300 °C, have a proven track record for backup power applications, but also represent a promising option for large‐scale stationary electricity storage due to the absence of critical raw materials, such as lithium and cobalt.^[^ ^1^, ^2^, ^3^, ^4^, ^5^, ^6^, ^7^, ^8^, ^9^, ^10^, ^11^, ^12^ ^]^ These batteries employ a solid ceramic Na‐*β*″‐alumina electrolyte in combination with a molten inorganic tetrachloroaluminate (NaAlCl_4_) electrolyte, improving operational safety compared to state‐of‐the‐art lithium‐ion batteries.^[^ ^13^, ^14^, ^15^ ^]^ However, while the energy density of sodium‐metal chloride batteries is relatively high (e.g., 140 Wh kg^−1^, 280 Wh L^−1^ on cell level), their charge and discharge rate capability needs to be improved to be competitive with lithium‐ion batteries.^[^ ^7^, ^16^ ^]^

Sodium‐metal chloride batteries are assembled in the discharged state. This avoids the handling of sodium metal, which forms the negative electrode, during cell assembly. Molten sodium is generated electrochemically during the first charge. Oxidation and reduction of sodium metal (Na ⇔ Na^+^ + e^−^) at temperatures above the melting point is extremely fast, enabling current densities beyond 1000 mA cm^−2^ without significant overpotential.^[^ ^17^ ^]^ It is thus the positive electrode, which limits the overall battery performance. The positive electrode is a composite, comprising mainly nickel, iron, and sodium chloride in the discharged state. The porous positive electrode is immersed in molten NaAlCl_4_ electrolyte, separated from the sodium metal negative electrode by the ceramic Na‐*β*″‐alumina electrolyte. During dis‐/charge cycling at 300 °C, the following half‐cell conversion reactions take place:^[^ ^1^, ^5^, ^6^, ^11^, ^18^ ^]^

Reduction/oxidation via de‐/chlorination of iron:

(1) $$\mathrm{Fe}+2\;\mathrm{NaCl}\Leftrightarrow \;\mathrm{FeC}l_{2}+2\mathrm{Na}\left(2.33V\mathrm{vs}\mathrm{Na}/{\mathrm{Na}}^{+}\right)$$

Reduction/oxidation via de‐/chlorination of nickel:

(2) $$\mathrm{Ni}+2\;\mathrm{NaCl}\Leftrightarrow \mathrm{NiC}l_{2}+2\mathrm{Na}\left(2.58V\mathrm{vs}\mathrm{Na}/{\mathrm{Na}}^{+}\right)$$

State‐of‐the‐art commercial sodium‐metal chloride cells derive the majority of their capacity from the de‐/chlorination of nickel,^[^ ^6^, ^19^ ^]^ which provides a higher cell potential and higher cycling stability compared to the de‐/chlorination of iron.^[^ ^20^ ^]^ During charge (1,2), sodium ions migrate from the positive to the negative electrode, and vice versa during discharge. Commercial cells feature a capacity of 38 Ah per cell, corresponding to an area specific capacity of approximately 150 mAh cm^−2^, and a porous positive electrode composite thickness of up to 18 mm,^[^ ^7^, ^21^ ^]^ supporting continuous dis‐/charging at 75 mA cm^−2^ (C/2).^[^ ^22^ ^]^ While pulsed dis‐/charge at higher current density is possible, the target for next‐generation sodium‐metal chloride batteries is 150 mA cm^−2^ continuous dis‐/charge (1C). For comparison, high‐energy lithium‐ion batteries feature capacities of only up to 4–5 mAh cm^−2^, and electrodes with a thickness on the order of only 0.1 mm, explaining their currently still superior rate capability.^[^ ^23^ ^]^

A recent study on sodium‐metal chloride cells identified the transport of Na^+^ ions as a rate‐limiting process in state‐of‐the‐art porous positive electrode composites, even for a relatively lower area specific capacity of 50 mAh cm^−2^.^[^ ^19^ ^]^ The self‐limiting nature of the conversion reaction of nickel and iron to nickel chloride and iron chloride causes local passivation.^[^ ^24^ ^]^ As a result, de‐/chlorination proceeds along a reaction front away from the Na‐*β*″‐alumina electrolyte, deeper into the positive electrode.^[^ ^21^ ^]^ Because iron is oxidized at a lower potential than nickel, the iron chlorination reaction front is spatially separated from (and precedes) the nickel chlorination reaction front. However, not all factors contributing to the dynamic evolution of cell resistance as a function of state of charge and dis‐/charge rate are yet understood.

Previously, the de‐/chlorination of various transition metals was studied by electrochemical model experiments in a common reservoir of molten NaAlCl_4_.^[^ ^20^, ^24^, ^25^, ^26^, ^27^, ^28^ ^]^ Diffusional rate limitations were identified to limit the reaction of nonporous iron and nickel foil or wire electrodes. Assuming de‐/chlorination to take place in a continuous, core–shell conversion layer, the transport limitations were assigned to diffusion of Cl^−^ or metal ions through the metal chloride conversion layer, either through pores,^[^ ^20^, ^24^, ^27^, ^28^ ^]^ or through the metal chloride itself.^[^ ^25^ ^]^ Limited reversibility in the oxidation and reduction of certain metal electrodes, as inferred from asymmetric current–voltage peak shapes in the model experiments, was ascribed to dissolution of cathode species.^[^ ^20^ ^]^ As a result, insolubility of metal chlorides and/or intermediate cathode species in the molten salt electrolyte (NaAlCl_4_) was defined as an essential requirement for successful sodium‐metal halide cathodes.^[^ ^29^ ^]^ However, the proposed solid‐state reaction mechanism conflicts with microstructural observations showing formation of metal chlorides in their particular crystal habit. It also struggles to account for the significant volume expansion inherent to the de‐/chlorination reactions (e.g., from 6.6 cm^3^ mol^−1^ for Ni to 36.5 cm^3^ mol^−1^ for NiCl_2_). Indeed, instead of a core–shell conversion layer, relatively large (5–10 µm) NiCl_2_ and FeCl_2_ crystals with lamellar and needle‐like morphology were identified in state‐of‐the‐art sodium‐metal chloride cathodes.^[^ ^30^ ^]^ Furthermore, model experiments with common NaAlCl_4_ reservoir suffer from unintended cross‐diffusion of dissolved metal species and their precipitation on the counter electrode, which can affect the assessment of kinetic parameters.^[^ ^27^, ^28^ ^]^

Thus, the aim of this study is to separate and isolate the different rate‐limiting processes which determine the cell resistance as a function of state of charge. In particular, we focus here on deconvoluting the de‐/chlorination processes of nickel and iron from microstructural effects of the porous electrodes (porosity, tortuosity, etc.). For the first time, we employ nonporous model electrodes in a realistic, planar cell geometry with separate electrode compartments. Combining electrochemical cell characterization with a detailed postmortem scanning electron microscopy (SEM) analysis of the electrodes, we identify the rate‐limiting process during de‐/chlorination and determine the diffusion coefficient of nickel and iron ions in molten NaAlCl_4_ electrolyte. These parameters can be employed directly in numerical models for optimizing the microstructure of the porous electrodes and derive guidelines for designing next‐generation sodium‐metal chloride batteries with improved rate performance.

## 2 Results and Discussion

**Figure 1:**
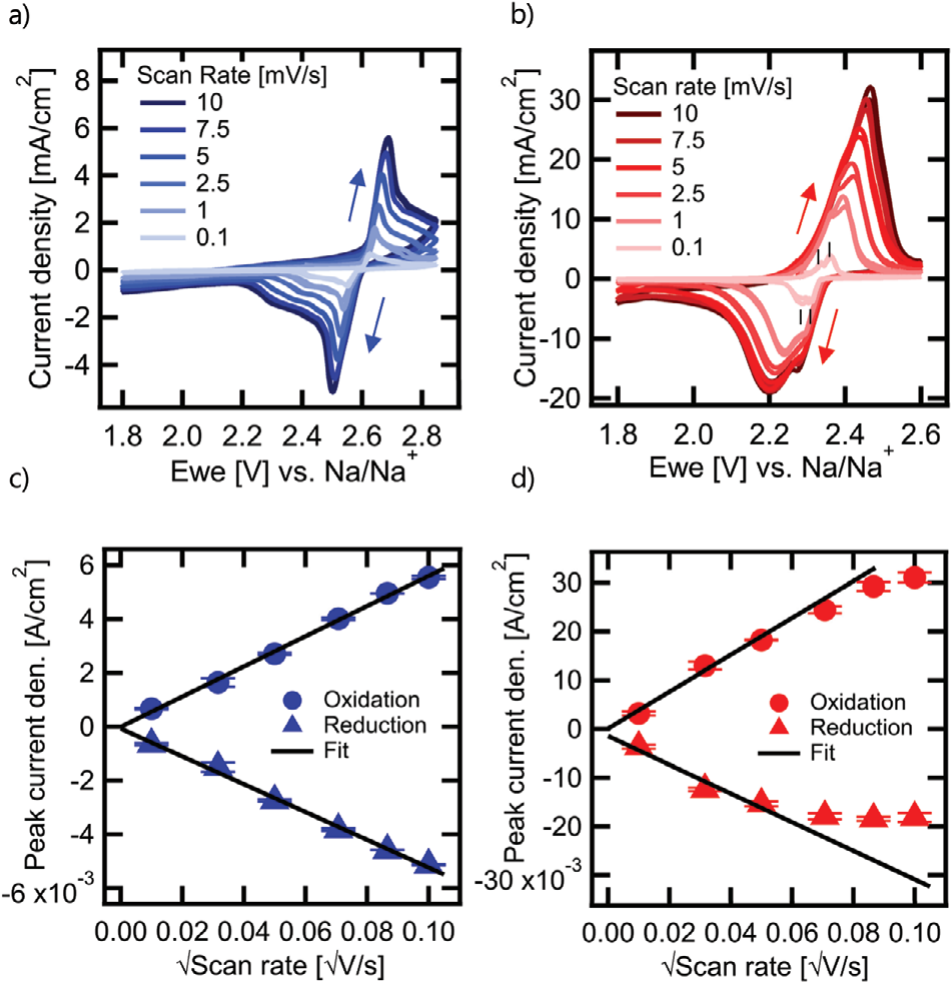
Cyclic voltammetry of a) Na/Ni‐NiCl_2_ and b) Na/Fe‐FeCl cells with planar Ni and Fe electrodes at scan rates from 0.1 to 10 mV s^−1^ and c,d) corresponding Randles–Sevcik plots.

In **Figure** **1**a, we show the cyclic voltammetry (CV) response of a Na/Ni‐NiCl_2_ cell with a planar nickel electrode for scan rates ranging from 0.1 to 10 mV s^−1^ at 300 °C. The voltage scan from 1.8 to 2.85 V and from 2.85 back to 1.8 V versus Na/Na^+^ results in a relatively symmetric current response centered around a voltage of 2.59 V versus Na/Na^+^ at 300 °C. The upper cut‐off voltage was limited to 2.85 V versus Na/Na^+^ to prevent decomposition of NaAlCl_4_ electrolyte at 3.05 V.^[^ ^31^ ^]^ Repeated cycling at a fixed scan rate within this voltage window results in a reversible current response (two overlapping voltage scans shown for each scan rate). At a scan rate of 10 mV s^−1^, oxidation and reduction current feature each a sharp current maximum at around 2.66 and 2.52 V, respectively, which move toward 2.59 V with decreasing scan rate (Figure S2a, Supporting Information). In scan direction, the current density rises rapidly toward the current maxima, but then falls offs relatively slowly, as de‐/chlorination proceeds at higher overpotentials, due to either 1) thickening of the chlorinated layer at the electrode surface and/or 2) reduced area of accessible unreacted electrode surface. Analysis of the CV response at 0.1 mV s^−1^ in a Tafel plot in Figure S2b, Supporting Information, allows estimation of the exchange current density, which is on the order of 0.3 mA cm^−2^. Identification of a linear regime in the Tafel plots is difficult, because the current density at this scan rate is still limited by cell resistance. Nevertheless, a reasonable estimate is obtained by setting the anodic transfer coefficient to 0.5, reflecting a reversible electrochemical reaction.^[^ ^32^ ^]^

Compared to the Na/Ni‐NiCl_2_ cell, the CV response for the Na/Fe‐FeCl_2_ cell shown in Figure 1b reveals much higher maximum current densities for the corresponding scan rates (note the different *y*‐axis scale). The upper voltage cut‐off was set to 2.6 V versus Na/Na^+^ to prevent the formation of FeCl_3_ species at 2.75 V.^[^ ^6^, ^25^ ^]^ The oxidation current density of the Fe‐FeCl_2_ electrode is dominated by a strong maximum consisting of two overlapping peaks, more clearly resolved at lower scan rates (up to 1 mV s^−1^, Figure S2c, Supporting Information). We attribute the first current peak at 2.34 V versus Na/Na^+^ to the formation of Na_6_FeCl_8_, and the second one at 2.36 V versus Na/Na^+^ to the formation of FeCl_2_, as previously reported.^[^ ^33^ ^]^ The oxidation and reduction current response is again relatively symmetric, centered around a voltage of 2.33 V versus Na/Na^+^ at 300 °C, and reversible at fixed scan rate (two overlapping voltage scans shown for each scan rate). In scan direction, the current density rises rapidly toward the current maxima, but then drops more rapidly than for the Na/Ni‐NiCl_2_ cell in both scan directions, indicating that de‐/chlorination proceeds possibly via a different mechanism (see discussion below). Analysis of the CV response at 0.1 mV s^−1^ in a Tafel plot in Figure S2d, Supporting Information, indicates an exchange current density, which is on the order of 3 mA cm^−2^, that is, a factor of 10 higher than for the Na/Ni‐NiCl_2_ cell, indicating a much faster de‐/chlorination mechanism.

In order to quantify the rate‐limiting process, current density maxima extracted from the CV‐scans in Figure 1a,b are plotted against the square root of the scan rate in Figure 1c,d. Due to the extremely high rate capability previously demonstrated for the sodium metal negative electrode in the same cell, but in symmetric electrode configuration,^[^ ^17^ ^]^ the peak current densities are solely assigned to the rate‐limiting process in the positive electrode. For a purely diffusion‐limited process, the maximum current densities obey Fick's laws of diffusion. The Randles–Sevcik equation represents a solution to Fick's second law for the special case when the concentration of the active species is zero at the electrode surface and constant in the electrolyte and predicts a linear relationship between maximum current density and the square root of the scan rate.^[^ ^34^ ^]^ Inspection of Figure 1c,d shows that the Na/Ni‐NiCl_2_ cell displays a very linear behavior for oxidation and reduction, while the Na/Fe‐FeCl_2_ cell displays a slight deviation from the linear behavior, especially during reduction, where maximum current densities stagnate above 5 mV s^−1^. The slopes of the Randles–Sevcik plot amount to 0.056 and −0.052 A cm^−2^ V^−0.5^ s^0.5^ for oxidation and reduction of nickel. With a nickel ion solubility of 2.1 × 10^−6^ mol cm^−3^ in NaAlCl_4_,^[^ ^35^, ^36^ ^]^ we can extract the diffusion coefficient of the rate‐limiting process in the Na/Ni‐NiCl_2_ cell, yielding 2.3 × 10^−3^ cm^2^ s^−1^ for oxidation and 1.9 × 10^−3^ cm^2^ s^−1^ for reduction. Details of the calculation are provided in the Supporting Information. Although the Randles–Sevcik plots of the Na/Fe‐FeCl_2_ do not exhibit a linear behavior over the full scan rate range, we get comparative values from a linear fit restricted to the lowest three scan rates(0.378 and −0.295 A cm^−2^ V^−0.5^ s^0.5^). This yields an upper bound for the diffusion coefficient of 1.2 × 10^−5^ cm^2^ s^−1^ during oxidation and 7.2 × 10^−6^ cm^2^ s^−1^ during reduction of iron, employing a value of 2.0 × 10^−4^ mol cm^−3^ for the iron ion solubility in NaAlCl_4_.^[^ ^33^, ^36^ ^]^ These upper bound values obtained for iron are more than two orders of magnitude smaller than the ones obtained for nickel! At first, this result seems counterintuitive, as the exchange current density estimated for iron is a factor of 10 higher than for nickel. This apparent contradiction can be reconciled by noting that the slope of the Randles–Sevcik equation shows a linear dependence on the concentration, but only a square root dependence on the diffusion coefficient (see equations in Supporting Information). Thus, our data shows that the faster de‐/chlorination in the iron system is due to the higher solubility of iron in NaAlCl_4_ compared to nickel, compensating the lower diffusion coefficient of iron ions compared to nickel ions in NaAlCl_4_ (or in the chloride layer).

**Figure 2:**
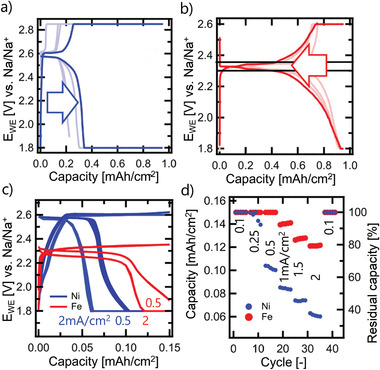
Evolution of cell voltage during galvanostatic run‐in cycling of the planar a) Ni‐NiCl_2_ and b) Fe‐FeCl_2_ electrode. c) Capacity limited cycling of planar Ni‐NiCl_2_ and Fe‐FeCl_2_ electrodes with 0.15 mAh cm^−2^, at discharge current densities of 0.5 and 2 mA cm^−2^, respectively. d) Rate test of planar Ni‐NiCl_2_ and Fe‐FeCl_2_ electrodes.

**Figure** **2**a,b compares galvanostatic dis‐/charge cycling of Na/Ni‐NiCl_2_ and Na/Fe‐FeCl_2_ cells with planar model electrodes at ±0.5 mA cm^−2^. When the upper cut‐off voltage of 2.85 and 2.6 V, respectively, is reached upon charge, the cells were held at this voltage until the current density dropped to 0.05 mA cm^−2^. Analogously, when the lower cut‐off voltage of 1.8 V is reached upon discharge, the cells were held at this voltage until the current density dropped to −0.05 mA cm^−2^. While the galvanostatically accessible discharge capacity increases slowly during the first four cycles for the Na/Ni‐NiCl_2_ cell from 0.22 to 0.35 mAh cm^−2^, the Na/Fe‐FeCl_2_ cell delivers a value of 0.95 mAh cm^−2^ already in the first cycle. This corresponds to the conversion of a ≈1.2 µm thick nickel or iron layer on the planar electrodes. For both types of model electrodes, the capacities accessed in these experiments substantially exceed the local degree of chlorination employed in porous high‐capacity cathodes. There, the cell voltage is maintained at a relatively low value, as the reaction front propagates deeper into the porous electrode, continuously accessing additional, unreacted electrode surface.

During galvanostatic charging, the cell voltage for the planar model electrode geometry increases due to either 1) thickening of the chlorinated layer at the electrode surface and/or 2) reduced area of accessible unreacted electrode surface. It is important to remind ourselves that for porous electrodes, the cell voltage can be maintained at a relatively low value, because 3) the reaction front propagates deeper into the porous electrode continuously “supplying” additional unreacted electrode surface. Consequently, in porous electrodes, the local degree of chlorination never reaches 0.95 mAh cm^−2^ per porous electrode area, which would lead to a breakdown of the electronic nickel/iron backbone of the porous electrode and rapid capacity fading due to loss of electronic contact of a fraction of the active electrode material to the current collector. Reversible, stable cycling of the planar model electrodes is achieved over 35 cycles in the rate‐test experiment when limiting the capacity to 0.15 mAh cm^−2^ (Figure 2d). This corresponds to an increase in cell voltage of ±0.03 V. Figure 2c compares galvanostatic dis‐/charge cycling of Na/Ni‐NiCl_2_ and Na/Fe‐FeCl_2_ cells with planar model electrodes and at a capacity limited to 0.15 mAh cm^−2^. During charge, a current density of 0.5 mA cm^−2^ was applied, while during discharge a current density of 0.5 and 2 mA cm^−2^ was employed. For each current density, five cycles are shown in Figure 2c exhibiting excellent cycling stability. Figure 2d shows data of a rate test for which the current density was increased from 0.1 to 2 mA cm^−2^. The rate test was concluded returning to 0.1 mA cm^−2^ resulting in the same capacity as during the first cycles at 0.1 mA cm^−2^, confirming that the electrodes do not degrade. While both electrodes are able to retrieve 100% of the initial capacity at 0.1 mA cm^−2^, this value drops to 81% for the Fe‐FeCl_2_ and to 41% for the Ni‐NiCl_2_ electrode at 2 mA cm^−2^. Thus, the rate tests confirms a significantly higher rate capability of the Fe‐FeCl_2_ electrode compared to the Ni‐NiCl_2_ electrode.^[^ ^20^, ^25^ ^]^

**Figure 3:**
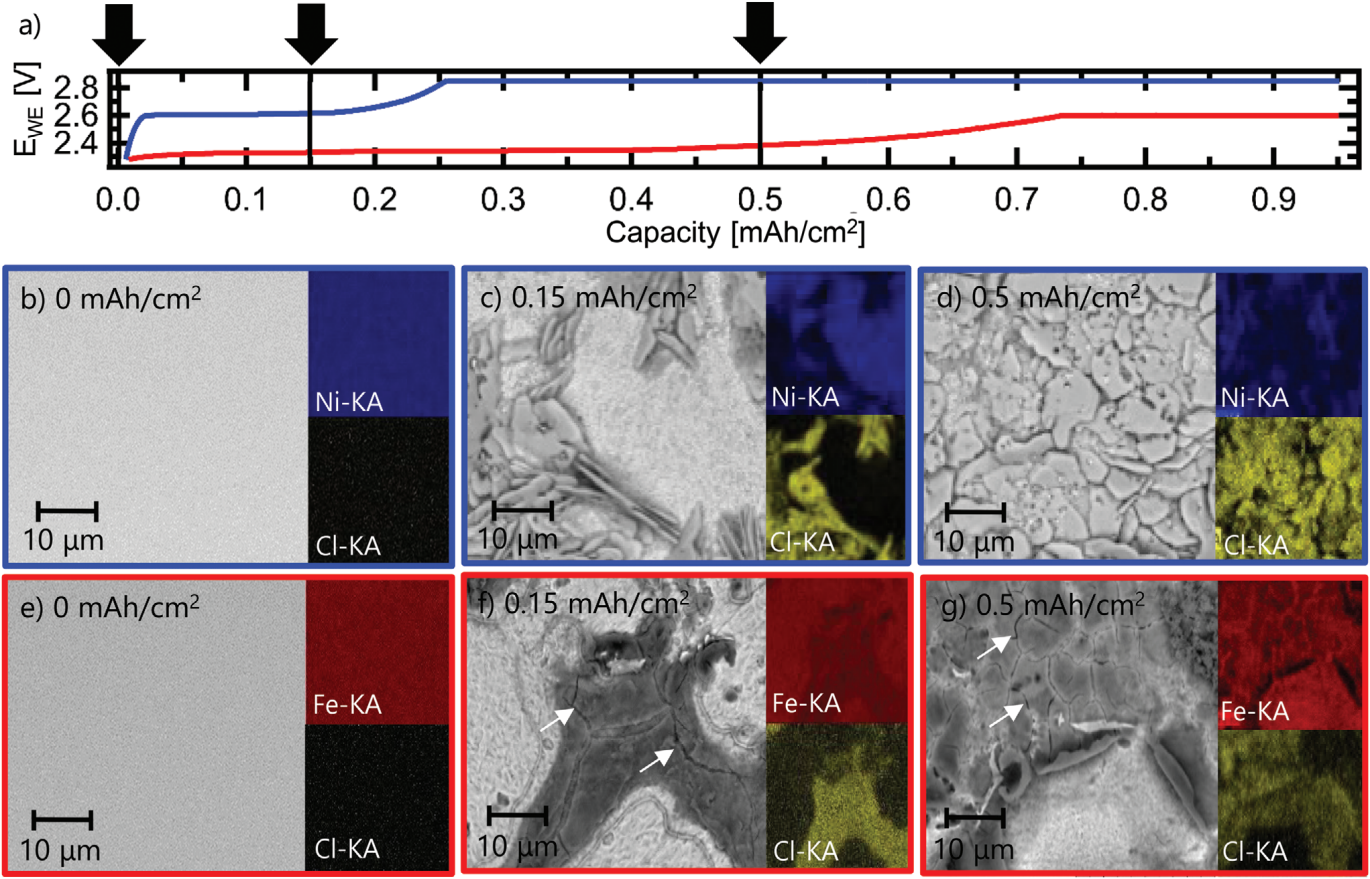
Postmortem analysis of Ni‐NiCl_2_ and Fe‐FeCl_2_ electrodes at different degrees of chlorination, after three cycles at 0.5 mA cm^−2^ and 300 °C. a) Charge curve for Ni‐NiCl_2_ (blue) and Fe‐FeCl_2_ (red) with arrows indicating the corresponding capacity at which the SEM images was taken. b) Pristine Ni electrode. c) Ni‐NiCl_2_ electrode at 0.15 mAh cm^−2^. d) Ni‐NiCl_2_ electrode at 0.5 mAh cm^−2^. e) Pristine Fe electrode. f) Fe‐FeCl_2_ electrode at 0.15 mAh cm^−2^. g) Fe‐FeCl_2_ electrode at 0.5 mAh cm^−2^.

**Figure** **3** links the electrochemical behavior of the Ni‐NiCl_2_ and Fe‐FeCl_2_ electrodes at a current density of 0.5 mA cm^−2^ to the corresponding surface morphology and composition at different degrees of chlorination. We performed SEM and energy‐dispersive X‐ray spectroscopy (EDS) mapping on electrodes extracted from disassembled cells after NaAlCl_4_ removal. Figure 3a shows the cell voltage of Na/Ni‐NiCl_2_ and Na/Fe‐FeCl_2_ cells during charge at 0.5 mAh cm^−2^, with arrows indicating the capacity at which the SEM images were taken. Comparison of Figure 3b,c shows that chlorination of the pristine, smoothly polished nickel electrode leads to immediate surface roughening and lateral heterogeneities already at 0.15 mAh cm^−2^. At this stage of chlorination, the surface of the nickel electrode features thin crystalline NiCl_2_ platelets (length 10–20 µm, thickness ≈1 µm) growing out of the electrode surface. The presence of crystalline NiCl_2_ is confirmed by the X‐ray diffraction (XRD) pattern of the Ni‐NiCl_2_ electrode shown in Figure S3, Supporting Information, consistent with the layered structure of the *R*‐3*m* space group.^[^ ^37^ ^]^ Thus, NiCl_2_ does not form in a conformal, core–shell structure on the nickel electrode, but segregates out of the molten NaAlCl_4_ electrolyte from dissolved nickel ions by nucleation and grain growth. At this degree of chlorination, the NiCl_2_ crystals do not yet fully cover the surface of the nickel electrode (≈50% surface coverage) as confirmed by the EDS maps in the inset. Full surface coverage is observed at 0.5 mAh cm^−2^ shown in Figure 3d. Enlarged nickel, chlorine, and sodium EDS maps are shown in Figure S4, Supporting Information. Upon discharge, NiCl_2_ crystals dissolve into the NaAlCl_4_ electrolyte. Nickel ions are plated back onto the electrode surface, resulting in a granular surface morphology with a typical feature size on the order of 1 µm (not shown).^[^ ^38^ ^]^ The remaining chlorine ions pair with Na^+^ to segregate NaCl crystals out of solution, which sediment on the nickel electrode surface (not shown).

The evolution of the iron electrode upon chlorination is shown in Figure 3e–g. In contrast to the nickel electrode, no particular crystal habit is observed for FeCl_2_ on the iron electrode surface. Instead we observe an unstructured FeCl_2_ conversion layer and a number of cracks on the electrode surface (white arrows in Figure 3f,g). The lower crystallinity is supported by the XRD pattern of the Fe‐FeCl_2_ electrode, showing broader FeCl_2_ peaks with lower signal to background ratio than those for Ni‐NiCl_2_ electrode (Figure S5, Supporting Information). After chlorination to 0.5 mAh cm^−2^, most of the iron electrode surface is converted to FeCl_2_, but along the localized attacks, there are still areas with nonconverted iron apparent in EDS maps of the iron electrode. These crack‐like structures in the iron electrode may form due to localized corrosion, resulting in the build‐up of stress caused by the formation of the FeCl_2_ layer.

**Figure 4:**
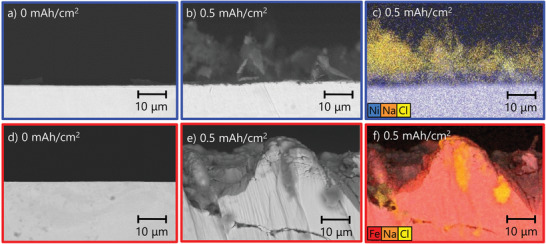
SEM images and EDS maps of ion‐milled cross sections of Ni‐NiCl_2_ and Fe‐FeCl_2_ electrodes. a) Pristine nickel electrode. b) Ni‐NiCl_2_ electrode at 0.5 mAh cm^−2^. c) EDS map of Ni‐NiCl_2_ electrode at 0.5 mAh cm^−2^. d) Pristine iron electrode. e) Fe‐FeCl_2_ electrode at 0.15 mAh cm^−2^. f) EDS map of Fe‐FeCl_2_ electrode at 0.5 mAh cm^−2^.

To confirm this hypothesis, we further investigated the chlorination behavior of the Ni‐NiCl_2_ and Fe‐FeCl_2_ electrode by imaging ion‐milled cross sections of the pristine and chlorinated electrodes as shown in **Figure** **4**a–c. After chlorination to 0.5 mAh cm^−2^, the Ni‐NiCl_2_ electrode maintains a relatively planar nickel surface with a surface roughness <1 µm, but grows a >10 µm thick layer of randomly oriented NiCl_2_ platelets on top. The EDS map in Figure 4c indicates that chlorination does not take place in the bulk of the nickel electrode. Separated Ni, Na, and Cl EDS maps can be found in Figure S6, Supporting Information. The situation is completely different for the Fe‐FeCl_2_ electrode, which exhibits a very rough electrode surface after chlorination to 0.5 mAh cm^−2^ with long cracks, penetrating deep into the electrode bulk (Figure 4d,e). The EDS map in Figure 4f confirms that chlorination does not take place in the bulk of the iron electrode, but consumes the iron electrode along the cracks, leading to the cracking off of larger iron pieces. This explains the poor cycling stability observed by earlier studies for planar and porous Fe‐FeCl_2_ electrodes, as electrode pulverization causes a loss of electronic contact of active electrode material to the current collector.^[^ ^20^, ^33^ ^]^ Separated Fe, Na, and Cl EDS maps can be found in Figure S7, Supporting Information.

**Figure 5:**
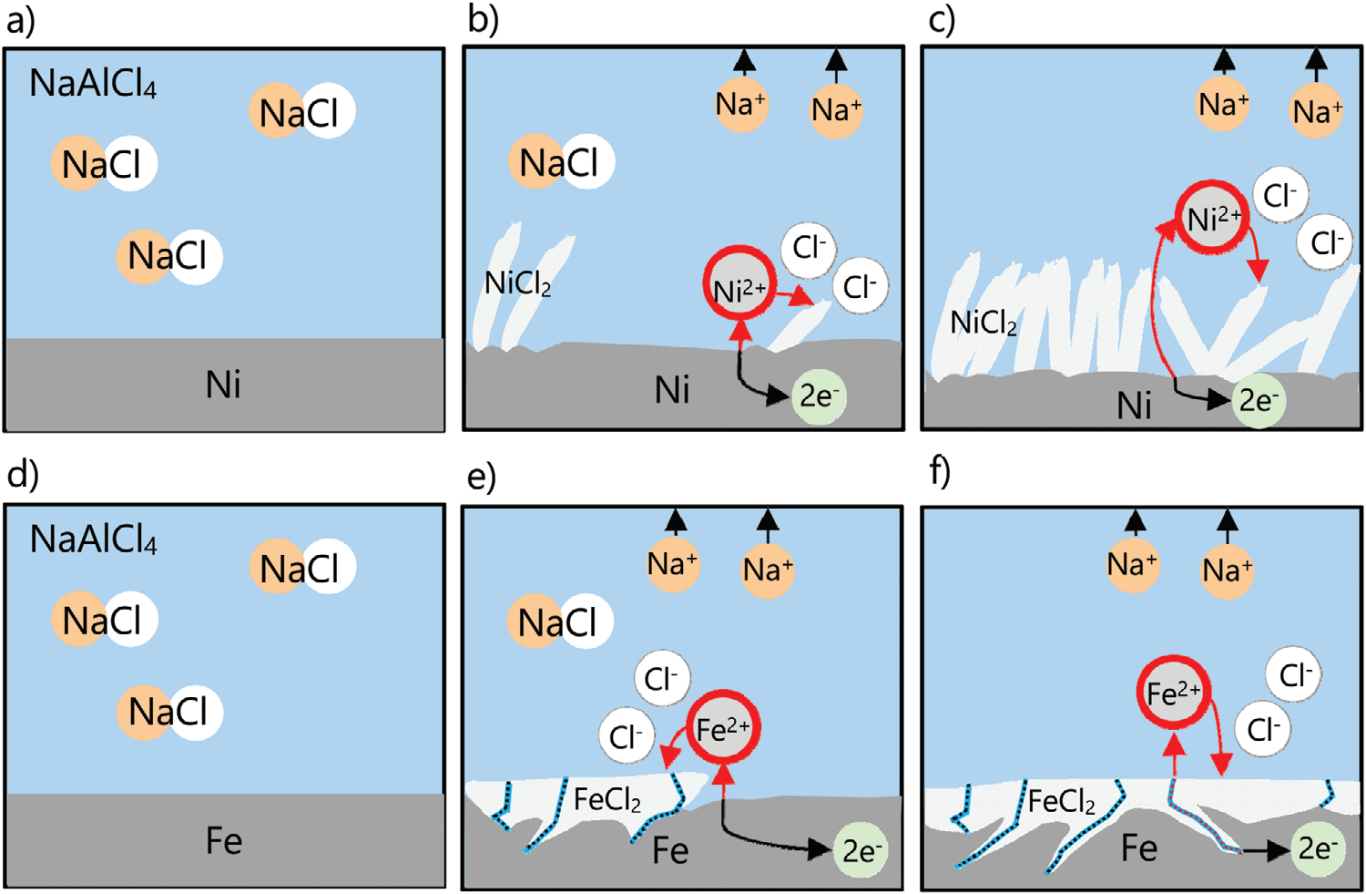
Sketch comparing chlorination of a–c) nickel and d–f) iron electrode at 0, 0.15, and 0.5 mA cm^−2^ state of charge.

In summary, our analysis shows that chlorination of both nickel and iron electrodes proceeds via dissolution and diffusion of metal cations through NaAlCl_4_. However, the nickel electrode promotes uniform oxidation of nickel and the formation of NiCl_2_ platelets (**Figure** **5**a–c). In contrast, localized corrosion processes (as identified in Figure 4e,f) and the higher solubility of iron ions in the NaAlCl_4_ electrolyte result in nonuniform iron oxidation and the pulverization of the iron electrode (see Figure 5d–f). Further investigations are required to determine whether formation of intermediate Na_6_FeCl_8_ affects this process (not shown here for simplicity). In any case, localized corrosion attack is a classical scenario, described also for many other electrode/electrolyte systems.^[^ ^39^, ^40^, ^41^ ^]^ Our analysis explains the faster dis‐/charge kinetics and higher rate capability observed for the iron electrode in Figures 1d and 2d as crack formation “supplies” continuously fresh iron surface for metal dissolution and chlorination as shown in Figure 5e,f.

Our results further demonstrate that metal‐ion diffusion and metal‐ion solubility in the molten NaAlCl_4_ electrolyte rather than transport across the metal chloride layer are limiting the rate capability of planar nickel and iron electrodes.^[^ ^25^ ^]^ Solubility of cathode species thus does not necessarily decrease the reversibility of the redox reactions in sodium‐metal chloride batteries. This has important implications for the design of porous high‐capacity electrodes. Increasing the mobility of metal ions improves peak current density only as the square root of the diffusion coefficient. It is thus more effective to increase metal‐ion solubility in the molten NaAlCl_4_ electrolyte, which improves peak current density linearly. However, increasing the metal‐ion solubility also leads to more rapid dissolution of the oxidized electrode, which can lead to a breakdown of the electronic backbone of the porous electrode and accelerate capacity fading due to loss of electronic contact of a fraction of the active electrode material to the current collector. Note that this phenomenon is independent of crack‐induced electrode pulverization, which represents an additional mechanism responsible for capacity fading in iron electrodes.

Metal‐ion diffusion and solubility become important, when the path for sodium ions is short and does not dominate the cell resistance and does not limit the current density. On charge, this is the case at low state of charge in porous electrodes, when the (oxidation) reaction front is situated in the vicinity of the Na‐*β*″‐alumina electrolyte. As the reaction fronts proceeds away from the Na‐*β*″‐alumina electrolyte during charging, the path length for sodium ions is increasing and consequently reaches a point, where the resistance contribution associated with the sodium‐ion transport will limit the current density. In this study, we cycled planar nickel electrodes at an areal capacity of 0.15 mAh cm^−2^, and observed rate limitations at current densities ≥0.25 mA cm^−2^ (Figure 2d). For comparison, a recent study on porous nickel cathodes demonstrated stable cycling for a capacity 50 mAh cm^−2^ at a current density of 160 mA cm^−2^, relative to the active cell area.^[^ ^19^ ^]^ With respect to the fine‐grained nickel surface (0.17 g cm^−2^ Ni255, surface area 0.64 m^2^ g^−1^), this corresponds to an areal capacity of 0.05 mAh cm^−2^, and continuous discharge at 0.15 mA cm^−2^, indicating that sodium ion transport limits thick porous nickel electrodes in these conditions. The effective path length for sodium ions strongly depends on the microstructure of the porous electrode (porosity, tortuosity, etc.). When designing the microstructure of a porous electrode, it is also important to take into account how the initial microstructure evolves during cycling (e.g., platelet versus crack formation). A multiphysics model based on an exact geometrical representation of the porous electrode would allow the design of tailored microstructures for different electrode compositions and dis‐/charge profiles.

On discharge, metal ions are plated onto the electrode. In principle, plating can result in local depletion of metal ions in the electrolyte, if metal chloride dissolution is not fast enough. However, because the values extracted from Figure 1c,d for the diffusion coefficients are comparable for oxidation and reduction, we conclude that the limiting factor is metal‐ion diffusion in the molten NaAlCl_4_ electrolyte rather than the rate of metal chloride dissolution.

Commercial sodium‐metal chloride batteries employ a combination of nickel and iron in the positive electrode to circumvent sodium‐ion transport limitations in the porous positive electrode, especially under pulsed current load. From our analysis, we can further conclude that addition of iron is useful to enhance the battery's rate capability by adding faster iron‐ion diffusion to the slower nickel‐ion diffusion. This is especially relevant at low state of charge when charging and high state of charge when discharging, when the reaction front is located in the vicinity of the Na‐*β*″‐alumina electrolyte and no sodium‐ion limitation is expected.

## 3 Conclusion

In summary, we reinvestigated de‐/chlorination of planar Ni‐NiCl_2_ and Fe‐FeCl_2_ electrodes in molten NaAlCl_4_ electrolyte. Despite the larger diffusion coefficient of nickel ions compared to iron ions in NaAlCl_4_, we observed a higher rate capability for the Fe‐FeCl_2_ electrode compared to the Ni‐NiCl_2_ electrode. This can be explained by the higher solubility of iron ions in NaAlCl_4_ and by localized corrosion attack in the iron electrode, continuously “supplying” fresh iron for chlorination. However, both factors also tend to accelerate capacity fading. We also observed that the nickel electrode is consumed uniformly and NiCl_2_ grows in platelets, while the iron electrode is consumed nonuniformly and FeCl_2_ growth as a layer.

Mechanistic insights into the de‐/chlorination of Ni‐NiCl_2_ and Fe‐FeCl_2_ electrodes gained in this study allow the design of improved positive electrode's with reduced charge and discharge times and therefore the competition with state‐of‐the‐art lithium ion batteries. This may hold the key for the breakthrough of sodium‐nickel‐chloride batteries for large‐scale electricity storage.

## 4 Experimental Section

A sketch and photographs of the self‐built high‐temperature cell used in the experiments are shown in Figure S1, Supporting Information. Na‐*β*″‐alumina disks with a thickness of 1 and 35 mm diameter were sintered at 1600 °C for 5 min from powders following ref. [42] and glued to two *α*‐alumina rings using a glass paste. To prevent dewetting of liquid sodium from the Na‐*β*″‐alumina surface on the negative electrode, a porous carbon coating was applied following the procedure described in ref. [17]. For the positive electrode, 20 mm diameter nickel (Ni201, 99.2%) and iron (Armco, 99.85%) were first polished (P2500, P4000) in isopropanol and then in inert argon atmosphere (P4000). 300 mg sodium NaAlCl_4_ (Sigma Aldrich, 99.99%) and 10 mg NaCl (JuraSel, 99.8%) powders were mixed to ensure NaCl saturation of the NaAlCl_4_ at all time. Full cells with an active area of 3.14 cm^2^ were assembled in an argon‐filled glovebox. A type K thermocouple was inserted at the interface between *α*‐alumina and Na‐*β*″‐alumina for precise temperature determination. Sodium metal foils with a purity of 99.9% and an area specific mass of 0.16 g cm^−2^ (0.5 g) were inserted at the negative electrode.

Cell heating for all experiments was realized via a resistive coil heater embedded in a self‐built glass fiber heating jacket. All experiments were conducted at 300 °C, while cell temperature was controlled via a feed‐forward controller. Cell cycling was conducted in a glovebox under argon atmosphere with H_2_O and O_2_ levels below 0.1 ppm. For CV measurements, each cell passed one sweep at each voltage scan rate for run‐in. For galvanostatic measurements prior to electrochemical and postmortem analysis, each cell passed three run‐in cycles at the corresponding area specific capacity (0.15, 0.5, 0.95 mAh cm^−2^) at 0.5 mA cm^−2^, with an upper cut‐off voltage at 2.85 V versus Na/Na^+^ for nickel, 2.6 V versus Na/Na^+^ for iron, 0.05 mA cm^−2^, and a lower cut‐off voltage of 1.8 V, 0.05 mA cm^−2^. During rate tests, the cells were charged (chlorinated) at 0.5 mA cm^−2^ up to 0.15 mAh cm^−2^ and discharged (de‐chlorinated) in the range of 0.1–2 mA cm^−2^. All cells were cycled with a Biologic VSP 3e potentiostat.

SEM images and EDS maps were taken after cell disassembly and removal of sodium tetrachloroaluminate (NaAlCl_4_) in isopropanol with a table top Hitachi TM3030Plus. XRD patterns were taken with a PANanalytical X`Pert PRO MRD. Cross sections of the electrodes were prepared by sawing and ion polishing employing a Hitachi IM4000 Plus ion miller.

## Conflict of Interest

The authors declare no conflict of interest.

## Supporting information

Supporting Information

## Data Availability

The data that support the findings of this study are available from the corresponding author upon reasonable request.
