# Supplementary material for: Elucidating the Rate‐Limiting Processes in High‐Temperature Sodium‐Metal Chloride Batteries
Source: Adv Sci (Weinh). 2022 Apr 11;9(17):2201019. doi: 10.1002/advs.202201019 (PMC9189643; doi:10.1002/advs.202201019)
Supplement: Supplementary file 1 — Supporting Information [file ADVS-9-2201019-s001.pdf]

ADVANCED SCIENCE
Open Access

Supporting Information

for *Adv. Sci.*, DOI 10.1002/advs.202201019

Elucidating the Rate-Limiting Processes in High-Temperature Sodium-Metal Chloride Batteries

Daniel Landmann, Enea Svaluto-Ferro, Meike V. F. Heinz, Patrik Schmutz and Corsin Battaglia*

Supporting Information

Elucidating the rate-limiting processes in high-temperature sodium-metal chloride batteries

Daniel Landmann[1,2]*, Enea Svaluto-Ferro*[1]*, Meike V. F. Heinz*[1]**, Patrik Schmutz*[1]*, Corsin Battaglia*[1]

[1]Empa, Swiss Federal Laboratories for Materials Science and Technology, 8600 Dübendorf, Switzerland

[2]Laboratory of Renewable Energy Science and Engineering, Ecole Polytechnique Fédérale de Lausanne, 1015 Lausanne, Switzerland

E-mail: meike.heinz@empa.ch

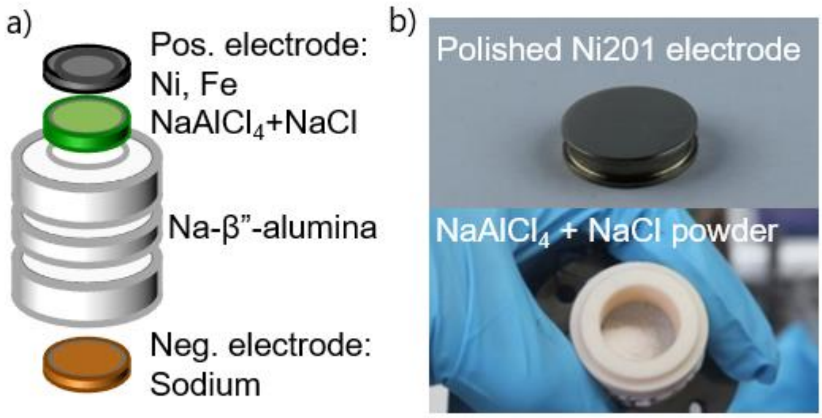

Figure S1. Electrochemical cell. a) Schematic image of cell. b) Image of pristine, polished nickel electrode and cell assembly filled with NaCl and TCA powder.

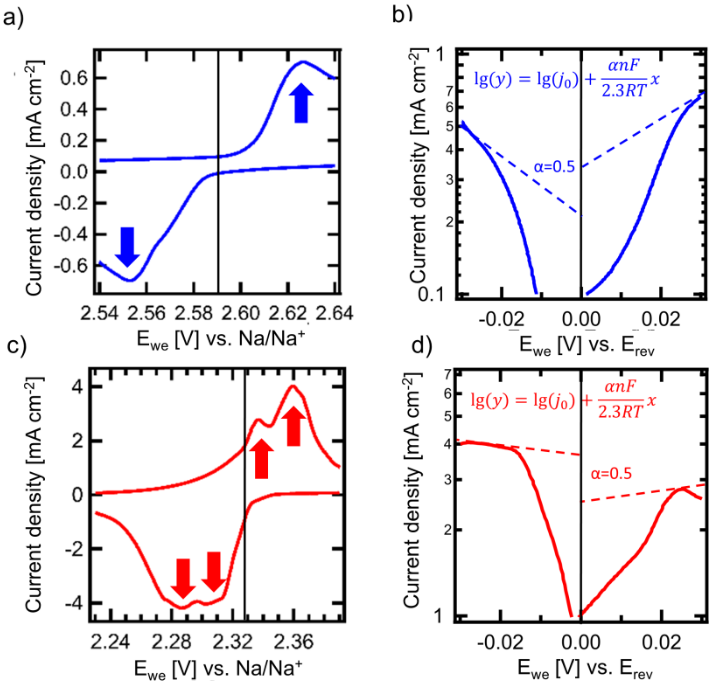

Figure S2. Cyclic voltammetry results at 0.1 mV s^{-1} for Ni-$NiCl_2$ (blue) and Fe-$FeCl_2$ (red). a) Current response of Na/Ni-$NiCl_2$ cell with single peaks upon oxidation and reduction with a reversible potential of E_{rev}= 2.59 V vs. Na/Na^+ at 300°C. b) Tafel-plot for Ni-$NiCl_2$ electrode with a reversible potential (E_{rev}) of 2.59 V vs. Na/Na^+. For reversible redox reactions, anodic and cathodic transfer coefficients are in the range of 0.4 – 0.6.[1] The dashed lines represent anodic and cathodic transfer coefficients of 0.5, which result in an exchange current density on the order of ~ 0.3 mA cm^{-2}. c) Current response of Na/Fe-$FeCl_2$ cell with two peaks upon oxidation and reduction with a reversible potential of E_{rev}= 2.33 V vs. Na/Na^+ at 300°C. d) Tafel-plot for Fe-$FeCl_2$ electrode with a reversible potential (E_{rev}) of 2.33 V vs. Na/Na^+. The dashed line represents an anodic and cathodic transfer coefficient of 0.5, resulting in an exchange current density of about ~ 3mA cm^{-2}.

Calculation of diffusion constants:

From Faulkner & Bard (pp.231)[2]:

$$i_{peak} = 0.4463(\frac{F^3}{RT})^{1/2} n^{\frac{3}{2}} A D_0^{\frac{1}{2}} c_0 \upsilon^{\frac{1}{2}}$$

$$j_{peak} = (0.4463(\frac{F^3}{RT})^{1/2} n^{\frac{3}{2}} D_0^{\frac{1}{2}} c_0) \cdot \upsilon^{\frac{1}{2}}$$

$$j_{peak} \propto |a| \cdot \upsilon^{\frac{1}{2}}\ ; |a| = \left(0.4463\left(\frac{F^3}{RT}\right)^{\frac{1}{2}} n^{\frac{3}{2}} D_0^{\frac{1}{2}} c_0\right) \rightarrow\ D_0 = \left(\frac{|a|}{0.4463\left(\frac{F^3}{RT}\right)^{\frac{1}{2}} n^{\frac{3}{2}}\ c_0}\right)^2$$

i_{peak}: Peak current [A]

j_{peak}: Peak current density [A cm^{-2}]

F: Faraday constant: 96485 [C mol^{-1}]

R: Universal gas constant: 8.314 [J molK^{-1}]

T: Temperature: 573.15 [K]

n: number of electrons involved: 2 [-]

D_0: Diffusion constant: [cm^2 s^{-1}]

c_0: initial concentration:[3, 4, 5] $c_{0,Ni2+}$ = 2.1544 e-6 [mol cm^{-3}]; $c_{0,Fe2+}$ = 1.9998 e-4 [mol cm^{-3}]: $\rho_{TCA,300°C}$=1.59985 g cm^{-3}

υ: scan rate: 0.0001-0.010 [V s^{-1}]

a: slope from Randles-Sevcik plot: a_{NiCl2} = $[\frac{A}{cm^2}\ (\frac{V}{s})^{-1/2}]$

$a_{Ni,ox}$:=0.056 $\frac{A}{cm^2}\ (\frac{V}{s})^{-1/2}$

$a_{Ni,\,red}$:=-0.052 $\frac{A}{cm^2}$ $(\frac{V}{s})^{-1/2}$

$a_{Fe,ox}$:=0.378 $\frac{A}{cm^2}$ $(\frac{V}{s})^{-1/2}$ (fit for first 3 points)

$a_{Fe,red}$:=-0.295 $\frac{A}{cm^2}$ $(\frac{V}{s})^{-1/2}$ (fit for first 3 points)

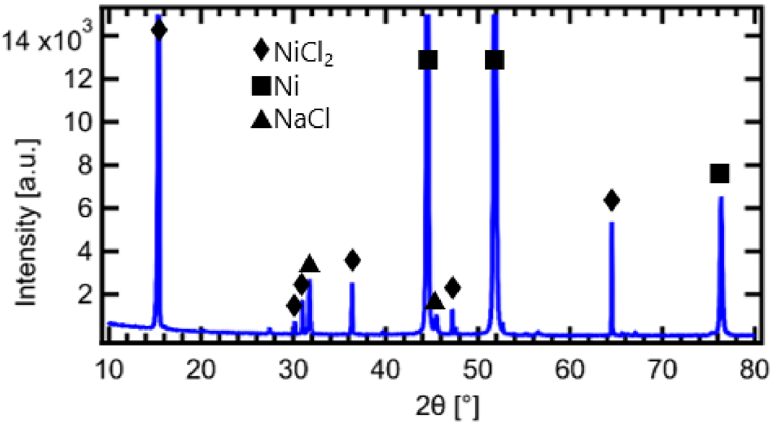

Figure S3. XRD pattern of Ni-$NiCl_2$ electrode at 0.5 mAh cm^{-2} showing the presence of NaCl, Ni and crystalline $NiCl_2$ (JCPDS: 01-071-2032).

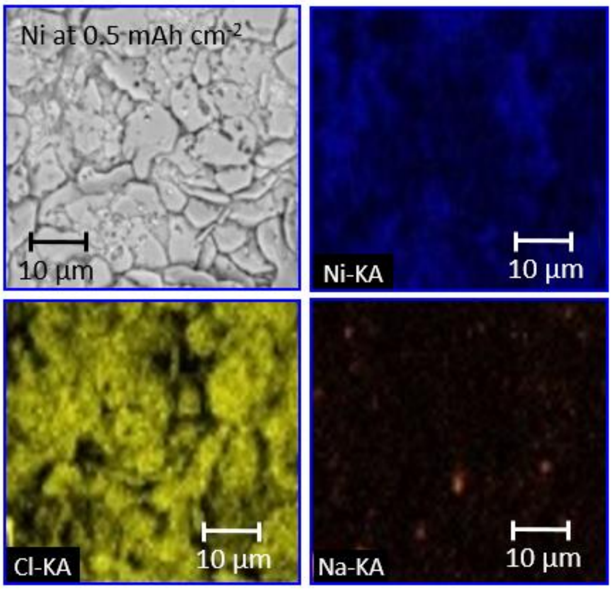

Figure S4. SEM image of nickel sample surface at 0.5 mAh/cm^2 with corresponding nickel, chlorine and sodium EDS maps, indicating the presence of nickel chloride platelets.

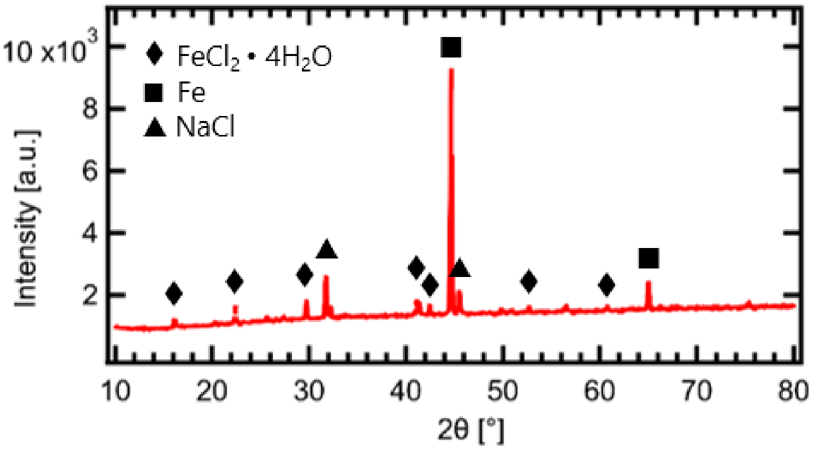

Figure S5. XRD pattern of Fe-$FeCl_2$ electrode at 0.5 mAh/cm^2 showing the presence of NaCl, Fe and amorphous, less-crystalline $FeCl_2 \bullet 4H_2O$.

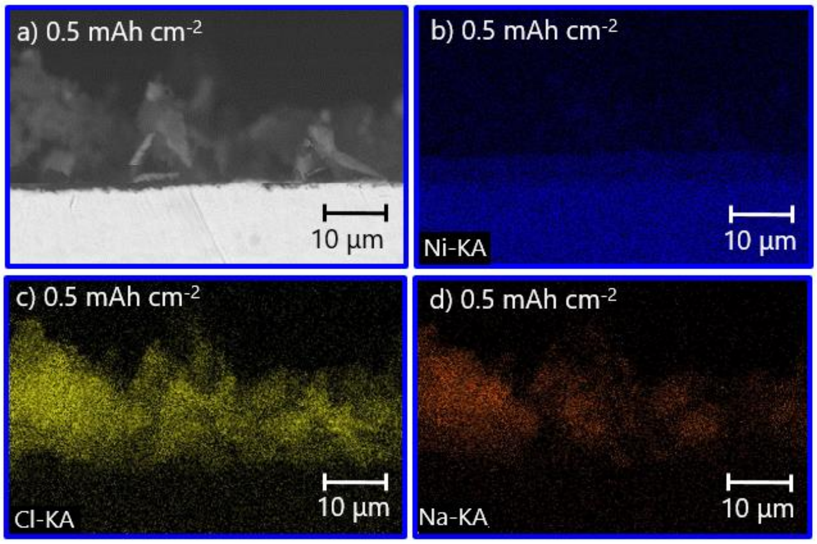

Figure S6. SEM image and EDS maps of ion-milled cross-sections of Ni-$NiCl_2$ electrode at 0.5 mAh cm^{-2}. a) SEM image. b) EDS Ni-KA. c) EDS Cl-KA. d) EDS Na-KA.

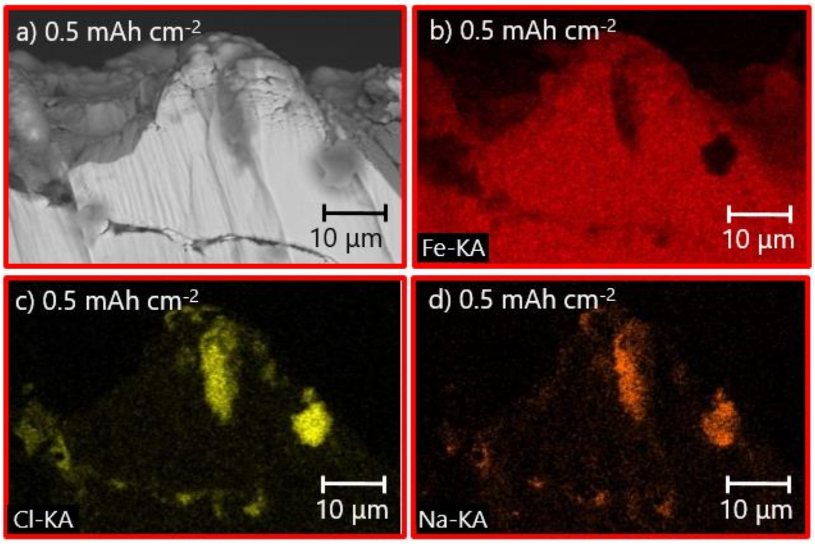

Figure S7. SEM image and EDS maps of ion-milled cross-sections of Fe-$FeCl_2$ electrode at 0.5 mAh cm^{-2}. a) SEM image. b) EDS Fe-KA. c) EDS Cl-KA. d) EDS Na-KA.

References

[1] C. H. Hamann, W. Vielstrich, Elektrochmie, Wiley-Vch Verlag Gmbh & Co KGaA; Weinheim, 2004.

[2] L. R. Faulkner, A. J. Bard, Electrochemical methods fundamentals and applications, Vol. 126, John Wiley & Sons, Inc, 2001.

[3] J. L. Coetzer, G. D. Wald, S. W. Orchard, J. Appl. Electrochem. 1993, 23, 790.

[4] M. G. Macmillan, B. Cleaver, J. Chem. Soc. Faraday Trans. 1993, 89, 3817.

[5] R. W. Berg, H. A. Hjuler, J. Niels, J. Chem. Eng. Data 1983, 28, 251.
